# Framework for denoising Monte Carlo photon transport simulations using deep learning

**DOI:** 10.1117/1.JBO.27.8.083019

**Published:** 2022-05-25

**Authors:** Matin Raayai Ardakani, Leiming Yu, David R. Kaeli, Qianqian Fang

**Affiliations:** a Northeastern University, Department of Electrical and Computer Engineering, Boston, Massachusetts, United States; b Analogic Corporation, Peabody, Massachusetts, United States; c Northeastern University, Department of Bioengineering, Boston, Massachusetts, United States

**Keywords:** Monte Carlo method, image denoising, photon transport, convolutional neural networks, deep learning

## Abstract

**Significance:**

The Monte Carlo (MC) method is widely used as the gold-standard for modeling light propagation inside turbid media, such as human tissues, but combating its inherent stochastic noise requires one to simulate a large number photons, resulting in high computational burdens.

**Aim:**

We aim to develop an effective image denoising technique using deep learning (DL) to dramatically improve the low-photon MC simulation result quality, equivalently bringing further acceleration to the MC method.

**Approach:**

We developed a cascade-network combining DnCNN with UNet, while extending a range of established image denoising neural-network architectures, including DnCNN, UNet, DRUNet, and deep residual-learning for denoising MC renderings (ResMCNet), in handling three-dimensional MC data and compared their performances against model-based denoising algorithms. We also developed a simple yet effective approach to creating synthetic datasets that can be used to train DL-based MC denoisers.

**Results:**

Overall, DL-based image denoising algorithms exhibit significantly higher image quality improvements over traditional model-based denoising algorithms. Among the tested DL denoisers, our cascade network yields a 14 to 19 dB improvement in signal-to-noise ratio, which is equivalent to simulating 25× to 78× more photons. Other DL-based methods yielded similar results, with our method performing noticeably better with low-photon inputs and ResMCNet along with DRUNet performing better with high-photon inputs. Our cascade network achieved the highest quality when denoising complex domains, including brain and mouse atlases.

**Conclusions:**

Incorporating state-of-the-art DL denoising techniques can equivalently reduce the computation time of MC simulations by one to two orders of magnitude. Our open-source MC denoising codes and data can be freely accessed at http://mcx.space/.

## 1 Introduction

Non-ionizing photons in the near-infrared (NIR) wavelength range have many benefits in biomedical applications compared with ionizing ones such as x-ray. Because of the low energy, NIR light is relatively safe to use and can be applied more frequently; the relatively low cost and high portability of NIR devices makes them excellent candidates for addressing needs in functional assessment in the bedside or natural environments.^1^ However, the main challenge of using low-energy NIR photons is the high degree of complex interactions with human tissues due to the presence of high scattering, which is much greater than that of x-rays. As a result, the success of many emerging NIR-based imaging or intervention techniques, such as diffuse optical tomography,^2^ functional near-infrared spectroscopy,^3^ photobiomodulation,^4^ etc., requires a quantitative understanding of such complex photon-tissue interactions via computation-based models.

The Monte Carlo (MC) method is widely regarded as the gold-standard for modeling photon propagation in turbid media,^5^ including human tissues, due to its accuracy and flexibility.^6^ It stochastically solves the general light propagation model—the radiative transfer equation (RTE)—without needing to build large simultaneously linear equations.^7^ As an approximation of RTE, the diffusion equation (DE) can be computed more efficiently using finite element-based numerical solvers,^8^ and DE is known to yield problematic solutions in regions that contain low-scattering media.^9^ In addition to the accuracy and generality, simplicity in implementing MC algorithms compared with other methods has made MC a top choice not only for teaching tissue-optics but also for developing open-source modeling tools.

MC methods have attracted even greater attention in recent years as simulation speed has increased dramatically due to the broad adoptions of massively parallel computing and graphics processing unit (GPU) architectures. The task parallel nature of MC algorithms allows it to be efficiently mapped to the GPU hardware.^10^ Current massively parallel MC photon propagation algorithms are capable of handling arbitrary 3D heterogeneous domains and have achieved hundreds-fold speedups compared with traditional serial simulations.^11^^–^^15^ This breakthrough in the MC algorithm has allowed biophotonics researchers to increasingly use it in routine data analyses, image reconstructions, and hardware parameter optimizations, in addition to its traditional role of providing reference solutions in many biophotonics domains.

A remaining challenge in MC algorithm development is the presence of stochastic noise, which is inherent in the method itself. Because an MC solution is produced by computing the mean behaviors from a large number of photon packets, each consisting of a series of random samplings of the photon scattering/absorption behaviors, creating high-quality MC solutions typically requires simulations of tens to hundreds of millions of photons. This number depends heavily on the domain size, discretization resolution, and tissue optical properties. This translates to longer simulation times because the MC runtime is typically linearly related to the number of simulated photons. From our recent work,^16^ a 10-fold increase of photon number typically results in a 10 decibel (dB) signal-to-noise ratio (SNR) improvement in MC solutions, suggesting that MC stochastic noise is largely shot-noise bound. From this prior work, we have also observed that the MC stochastic noise is spatially varying and, in highly scattering/absorbing tissues, exhibits a high dynamic range throughout the simulation domain.

To obtain high-quality simulation results without increasing the number of simulated photons, signal processing techniques have been investigated to remove the stochastic noise introduced by the MC process. This procedure is commonly referred to as denoising.^16^^,^^17^ In the past, model-based noise-adaptive filters have been proposed to address the spatially varying noise in the radiation dosage estimation context and computer graphics rendering.^18^^–^^20^ However, improvements provided by applying these filtering-based techniques have been small to moderate, creating an equivalent speedup of only three- to fourfold.^16^ Recent works on denoising ray-traced computer graphics and spatially variant noisy images in the field of computer vision focus mainly on machine-learning (ML)-based denoising methods, more specifically convolutional neural networks (CNNs).^17^ Despite their promising performance compared with traditional filters, no attempt has been made, to the best of our knowledge, to adapt denoisers designed for the two-dimensional (2D) low bit-depth image domain to high dynamic range MC fluence maps.^16^^,^^21^ Our motivation is therefore to develop effective CNN-based denoising techniques, compare them with state-of-the-art denoisers in the context of MC photon simulations, and identify their strengths compared with traditional model-based filtering techniques.

In recent years, the emergence of CNNs has revolutionized many image-processing-centered applications, including pattern recognition, image segmentation, and super-resolution. CNNs have also been explored in image denoising applications, many targeted at removing additive white Gaussian noise (AWGN) from natural images^22^ and, more recently, real camera noise.^23^^,^^24^ Compared with classical approaches, CNNs have also demonstrated impressive adaptiveness to handle spatially varying noise.^25^^,^^26^ In a supervised setting, given a dataset representative of media encountered in real-life simulations, CNNs have shown to better preserve sharp edges of objects without introducing significant bias, compared with model-based methods.^22^^,^^27^^,^^28^ Finally, due to extensive efforts over the past decade to accelerate CNNs on GPUs, modern implementations of CNN libraries can readily take advantages of GPU hardware to achieve high computational speed compared with traditional methods. Nonetheless, there has not been a systematic study to quantify CNN image denoiser performance over MC photon transport simulation images, either in 2D or 3D domains.

The contributions of this work are the following. First, we develop a simple generative model that uses the Monte Carlo eXtreme (MCX)^12^ software to create a synthetic dataset suited for supervised training of an image denoiser, providing ample opportunities for learning its underlying noise structure. Second, we develop and characterize a novel spatial-domain CNN model that cascades DnCNN^26^ (an effective global denoiser) and UNet^29^ (an effective local denoiser). Third, we adapt and quantitatively compare a range of state-of-the-art image denoising networks, including DnCNN,^26^ UNet,^29^ DRUNet,^28^ deep residual-learning for denoising MC renderings^30^ (referred to as ResMCNet hereinafter), and our cascaded denoiser, in the context of denoising 3D MC simulations. We assess these methods using a number of evaluation metrics, including mean-squared error (MSE) and structural similarity index measure (SSIM). For simplicity, other deep-learning (DL)-based denoising methods that do not operate in the spatial domain^31^^,^^32^ or require specialized knowledge from their target domain^33^ are not investigated here and are left for future work. Finally, a range of challenges encountered during the development of our approach are also discussed, providing guidance to future work in this area.

## 2 Methods

### 2.1 Training Dataset Overview

To train and evaluate CNN denoisers in a supervised fashion, a series of datasets that provided one-to-one mappings between “noisy” and “clean” simulations were generated. The training dataset was created using our MCX software package,^12^ in which simulations of a range of configurations with different photon levels were included. The 3D fluence maps generated from the highest number of photons were treated as clean data, and the rest were regarded as noisy. For this work, all configurations were simulated with photon numbers between $10^{5}$ and $10^{9}$ with a 10-fold increment. Simulations with $10^{9}$ photons were selected as the “ground truth,” because they provide the closest estimate to the noise-free solutions. Therefore, the CNN denoisers are tasked to learn a mapping between simulations with photon numbers lower than $10^{9}$ to results simulated with $10^{9}$ photons.

#### 2.1.1 Generation of training and validation datasets

**Fig. 1:**
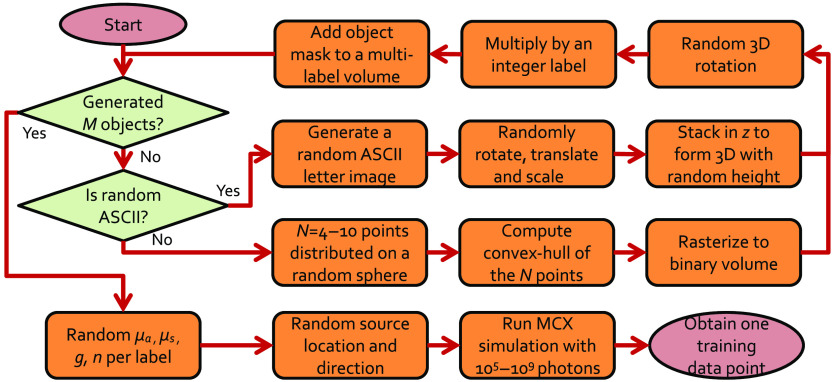
Workflow diagram for creating random simulation domains for the training/validation data.

To efficiently generate a large and comprehensive corpus of representative MC training data, first a volume generation scheme was designed. In such a scheme, arbitrarily-shaped and -sized polyhedrons and random 3D American standard code for information interchange (ASCII) characters with arbitrary sizes are randomly placed inside a homogeneous background domain with random optical properties. Using combinations of ASCII characters and polyhedrons produces a wide variety of complex shapes, while keeping the data generation process efficient. A similar letter-based random domain generation approach has been previously reported for training networks for fluorescence lifetime imaging.^34^ A diagram showing the detailed steps for creating a random simulation domain for generating training data is shown in Fig. 1.

Specifically, a random number (M=0 to 4) of randomly generated shapes, either in the form of 3D polyhedrons or 3D ASCII letters, are first created as binary masks, with the same size as the target volume. Then, the binary mask is multiplied by a label—a unique identification number assigned to each object—and subsequently accumulated in a final volume, in which voxels marked with the same label belong to the same shape. In the process of accumulation and generation of binary masks for each shape, if two or more objects intersect, this process creates new inclusions for the overlapping regions. We generate all training datasets on 64×64×64 (in $1\text{-}{\mathrm{mm}}^{3}$ isotropic voxels) domains, while the datasets for validation are 128×128×128 voxels. This allows us to observe the scalability of the networks to volume sizes that are different than the training dataset. A total of 1500 random domains are generated for training and 500 random domains for the “validation.” During training, the average global metrics (explained in Sec. 2.4.1) of the model computed over the validation dataset are saved over single epoch intervals. At the end of the training, the model with the best overall metrics is selected as the final result.

To create random 3D polyhedrons, a number of points (N=4 to 10) are determined on a sphere of random location and radius using the algorithm provided by Deserno.^35^ The convex-hull of the point set is computed and randomly rotated and translated in 3D. This convex-hull is subsequently rasterized into a binary mask.

For ASCII character inclusions, first, a random character in either lower or upper cases of English alphabet is selected. A random font size is chosen from a specified range, and the letter is rendered/rasterized in a 2D image with a random rotation angle and position. This binary 2D mask is further stacked with a random thickness to form a 3D inclusion. Finally, a 3D random rotation/translation is applied to the 3D ASCII character inclusion.

After generating a random volume, a random simulation configuration is generated to enable simulations with MCX. This includes determining the optical properties, including the absorption ($\mu_{a}$), scattering ($\mu_{s}$) coefficients, anisotropy (g), and refractive index (n), for each of the labels inside the generated volume, as well as the light source position and launch direction for the simulation. For the training and validation datasets, only isotropic sources are used for simplicity. The source is randomly positioned inside the domain.

**Fig. 2:**
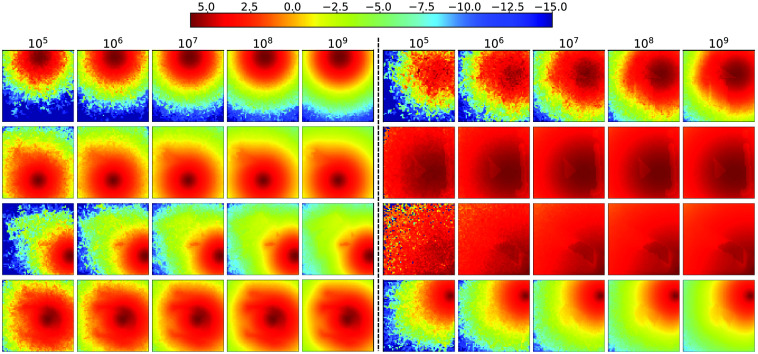
Sample MC fluence images (slices from 3D volumes) generated for CNN training.

The random optical properties are determined in ranges relevant to those of biological tissues, including (1) $\mu_{a}=|N(0.01;0.05)|\;{\mathrm{mm}}^{-1}$, where N(μ;σ) is a normal distribution with mean μ and standard deviation σ; (2) g is a uniform random variable between 0.9 and 1, (3) $\mu_{s}=\mu_{s}^{\prime }/(1-g)$, where the reduced scattering coefficient $\mu_{s}^{\prime }=|N(1;1)|\;{\mathrm{mm}}^{-1}$; and 4) n is a uniformly distributed random variable between 1 and 10. For all data, we simulate the continuous-wave fluence for a time-gate length randomly selected between 0.1 and 1 ns. Each simulation uses a random seed. In Fig. 2, we show a number of image slices (log-10 scale) from 3D simulation samples ranging from homogeneous domains to heterogeneous domains containing multiple polyhedral or letter-shaped inclusions.

#### 2.1.2 Data augmentation

To increase the diversity of the generated dataset and avoid overfitting, data augmentation^36^ was used. Our data augmentation consisted of 90-deg rotation and flipping. Each transformation was applied independently over a randomly selected axis. Transforms were identically applied to both inputs and labels of the training data. Both transforms were randomly selected and applied, with a probability of 0.7. This on-the-fly strategy multiplied the data encountered by the models during training by 256 without performing any time-consuming MC simulation.

#### 2.1.3 Test datasets

Three previously used standard benchmarks,^16^ (B1) a $100\times 100\times 100\;{\mathrm{mm}}^{3}$ homogeneous cube with a 1-mm voxel size, (B2) the same cubic domain with a $40\times 40\times 40\;{\mathrm{mm}}^{3}$ cubic absorber, and (B3) the same cubic domain with a refractive inclusion, were employed to characterize and compare the performance of various denoising methods. The optical properties for the background medium, the absorbing and refractive inclusions, can be found in Sec. 3 of our previous work.^16^ Each of the benchmarks was simulated with 100 repetitions using different random seeds. Additionally, the Colin27^12^^,^^37^ atlas (B4), Digimouse^38^ atlas (B5), and University of Southern California (USC) 19.5^39^ atlas (B6) from the Neurodevelopmental MRI database^40^ were selected as examples of complex simulation domains to test our trained MC denoisers. In addition, we also included a benchmark (B7) containing a ball-lens to test the performance of our denoisers in low-albedo media. In this benchmark, a cubic domain of 100×100×100 grid of $0.1\;{\mathrm{mm}}^{3}$ isotropic voxels is filled with medium of $\mu_{a}=0.01/\mathrm{mm}$, $\mu_{s}=1/\mathrm{mm}$, g=0.95, and n=1. A spherical-lens of 2-mm radius is placed in the center of the domain and filled with a medium of $\mu_{a}=0.01/\mathrm{mm}$, $\mu_{s}=1/\mathrm{mm}$, g=0.9, and n=1.4. A pencil beam pointing toward the +z axis is located at (4, 5, 0) mm. The domain volume is pre-processed to utilize the split-voxel MC algorithm,^41^ which can accurately handle curved media boundaries rasterized using a voxelated domain.

### 2.2 Pre-Processing of Monte Carlo Data

Many of the reported DL denoising techniques were developed to process natural images of limited bit-depth that usually do not present the high dynamic range as in MC fluence maps. To allow CNNs to better recognize and process unique MC image features and avoid difficulties due to limited precision, we applied the following transformation to the fluence images before training or inference:

y=t(x)=ln(c×x+1), (1)

where x is the MC fluence map, c is a user-defined constant, and the output y serves as the input to the CNN. This transformation serves two purposes. First, it compresses the floating-point fluence values to a limited range while equalizing image features across the domain. Second, it compensates for the exponential decay of light in lossy media and reveals image contracts that are relevant to the shapes/locations of the inclusions, assisting the CNN to learn the features and mappings. The addition of 1 in Eq. (1) ensures that t(x) does not contain negative values. An inverse transform $t^{-1}(y^{\prime })=(e^{y^{\prime }}-1)/c$ is applied to the output of the CNN ($y^{\prime }$) to undo the effect of this transform.

Moreover, when training a CNN on 8-bit natural image data, a common practice is to divide the pixel values by the maximum value possible (i.e., 255) to normalize the data. From our tests, applying such an operation on floating-point fluence maps resulted in unstable training; therefore our training data were not normalized.

Additionally, due to limited data precision, we noticed that all tested CNN denoisers exhibit reduced denoising image quality when processing voxel values (before log-transformation) that are smaller than an empirical threshold of 0.03. To address this issue and permit a wider input dynamic range, two separate copies of the fluence maps were denoised during inference—the first copy was denoised with c set to 1 and the second one with c set to $10^{7}$. The final image is obtained by merging both denoised outputs: voxels that originally had fluence values larger than 0.03 retrieve the denoised values from the first output and the rest are obtained from the second output. This variable-gain approach allowed us to process MC fluence images containing both high and low floating-point values.

### 2.3 Cascaded MC Denoising Network that Combines DnCNN and UNet Networks

**Fig. 3:**
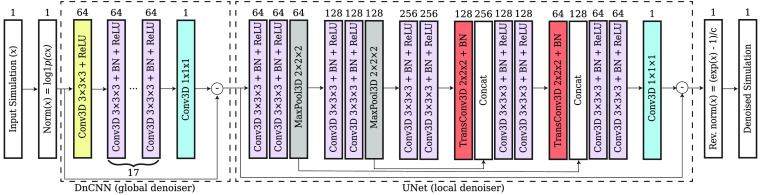
Overview of the cascaded DnCNN + UNet architecture. Each block in the dashed squares represents a group of CNN layers that are applied sequentially. The number on the square block indicates the number of channels for the respective output tensor. Conv3D, TransConv3D, and BN stand for 3D convolution, 3D transposed convolution, and batch normalization layers, respectively. PyTorch function log1p(cx) is a stable implementation of function ln(cx+1).

In this work, we designed a cascaded CNN denoiser, as shown in Fig. 3, specifically optimized for denoising our 3D MC fluence maps by combining two existing CNN denoisers: a DnCNN denoiser is known to be effective for removing global or spatially invariant noise, especially AWGN, without any prior information,^26^ whereas a UNet denoiser is known to remove local noise that is spatially variant.^28^^,^^29^ Therefore, in our cascaded DnCNN/UNet architecture, referred to as “cascade” hereinafter, the CNN first learns the global noise of an MC fluence image and attempts to remove it. The remaining spatially variant noise can then be captured and removed using a UNet. In both stages, the noise is learned in the residual space, meaning that, instead of mapping a noisy input to a clean output directly, the network maps the noisy input to a noise map and then subtracts it from the input to extract the clean image.

We want to mention that cascaded denoisers similar to the above design have been proposed for processing real-world images.^25^^,^^42^ In these works, a model-based filter (BM3D) serves as the global denoiser to provide an improved prior to a CNN-based local denoiser. In comparison, our method utilizes CNN denoisers for both global and local denoising stages, making it possible to train and accelerate fully on GPUs while automatically adapting to varying levels of noise.

### 2.4 Denoising Performance Metrics

#### 2.4.1 Global performance metrics

The global resemblance between the denoised volume and the ground truth (in this case, simulations with $10^{9}$ photons) can be used to measure the performance of a denoiser. A number of metrics measuring such a similarity have been used by others to evaluate image restoration networks or measure convergence.^21^^,^^26^^,^^43^^,^^44^ Typically, these metrics are defined for 2D images; in this work, we extended the definitions to apply to 3D fluence maps.

The most commonly used objective functions for denoising networks are the mean least squared error ($L_{2}$) and mean absolute error ($L_{1}$):

$$L_{n}(\theta )=\frac{1}{K}\overset{K}{\underset{i=1}{\sum }}{\left|F(y_{i};\theta )-x_{i}\right|}^{n},$$ (2)

where K is the number of noisy-clean fluence map pairs sampled from the dataset, referred to as the “mini-batch” size; θ contains all parameters of the network; F denotes the network itself; n is either 1 or 2; and $\left(x_{i},y_{i}\right)$ denotes the i’th noisy-clean pair of data in the mini-batch. These error metrics are widely used in supervised denoising networks, including the DnCNN, DRUNet, and ResMCNet models, as well as several other studies.^25^^,^^26^^,^^28^^,^^30^^,^^42^ $L_{1}$ and $L_{2}$ may have different convergence properties.^43^ The $L_{1}$ loss has gained more popularity in the DL community due to its good performance and low computational costs.^30^^,^^43^ For this work, however, to penalize large errors more, the $L_{2}$ loss was used instead to train the networks.

In contrast to $L_{n}$ distances, SSIM^45^ provides a perceptually motivated measure that emulates human visual perception for images. The SSIM for a pixel in an image is defined as

$$\mathrm{SSIM}(p)=\frac{2\mu_{x}\mu_{y}+C_{1}}{\mu_{x}^{2}+\mu_{y}^{2}+C_{1}}\times \frac{2\sigma_{xy}+C_{2}}{\sigma_{x}^{2}+\sigma_{y}^{2}+C_{2}},$$ (3)

where $\mu_{x}$ and $\sigma_{x}$ are the mean and standard deviation of the image x, respectively, and $\sigma_{xy}$ is the co-variance of images x and y. The statistics are calculated locally by convolving both volumes with a 2D Gaussian filter with $\sigma_{G}=5$. Small constants $C_{1}$ and $C_{2}$ are used to avoid division by zero. The SSIM value of two images is the average SSIM computed across all pixels, with a value of 1 suggesting that the two images are identical, and a value of 0 suggesting that the two images are not correlated. This definition can also be applied to 3D fluence maps using a 3D Gaussian kernel to calculate neighborhood statistics.

Another metric, peak signal-to-noise ratio (PSNR), measures the ratio between the maximum power of a signal and the power of the noise.^46^ The PSNR for two volumes x and y is expressed as

$$\mathrm{PSNR}(x,y)=20\;\log_{10}(\frac{I_{\max}}{\sqrt{{\left\|x-y\right\|}_{2}}}).$$ (4)

Larger PSNR values indicate smaller $L_{2}$ distances between volumes. The $I_{\max}$ value is the maximum value that a voxel can have in a fluence map after the transformation in Eq. (1). Therefore, in this work, we set $I_{\max}$ to 40.

#### 2.4.2 Local performance metrics

A number of locally (voxel-bound) defined performance metrics have been used in our previous MC denoising work.^16^ The SNR of the denoised volumes for each voxel measures the efficacy of the denoiser of spatially adaptive noise. For a simulation running k photons, we first run multiple (N=100) independently seeded MC simulations and compute SNR in dB with

$${\mathrm{SNR}}_{k}(r)=20\;\log_{10}\;\frac{\mu_{k}(r)}{\sigma_{k}(r)},$$ (5)

where $\mu_{k}$ and $\sigma_{k}$ are the mean and standard deviation of voxel values at location r across all repetitions, respectively. The average SNR difference before and after applying the denoising filter, ΔSNR, is subsequently calculated along selected regions of interest.

Our previous work^16^ suggests that the noise in MC images largely follows the shot-noise model; therefore, increasing the simulated photon number by a factor of 10 results in ∼10  dB improvement in SNR on average. We have previously proposed a photon number multiplier^16^ $M_{F}$ to measure equivalent acceleration using the average SNR improvement ΔSNR:

$$M_{F}=10^{\frac{\Delta \mathrm{SNR}}{10}}.$$ (6)

For example, a ΔSNR=20  dB gives $M_{F}=100$, suggesting that the denoised result is equivalent to a simulation with 100 times the originally simulated photon number, which is equivalent to accelerating the simulation by a factor of 100 if the denoising run-time is ignored.

### 2.5 Implementation Details

#### 2.5.1 BM4D and ANLM

Block-matching four-dimensional collaborative filtering (BM4D) and our GPU-accelerated adaptive non-local means (ANLM)^16^ are used as representative state-of-the-art model-based denoisers and used to compare against CNN-based denoisers. For BM4D, a Python interface developed based on the filter described by Mäkinen et al.^47^ was used, whereas for the ANLM filter, a MATLAB function developed previously by our group^16^ was used.

#### 2.5.2 CNN training details

All CNN denoising networks were re-implemented for handling 3D data using the open-source DL framework, PyTorch.^48^ For most of the studied CNN denoisers, our implementations largely follow their originally published specifications but replacing the 2D layers with their 3D variants. Small adjustments were made. For UNet, for example, 3D batch normalization (BN) layers were introduced in between the 3D convolution, the convolution transpose, and the pooling layers to address the covariance shift problem.^49^ Additionally, we simplified ResMCNet by removing the auxiliary features needed for computer graphics renderings purposes, making the kernel size of the first layer 3 instead of 7.

All networks in this study were trained for 1500 epochs on a single NVIDIA DGX node equipped with eight NVIDIA A100 GPUs, each with 40 GB of memory and NVLink 2.0 connection. Leveraging the PyTorch scaling wrapper, PyTorch Lightning^50^ was used to simplify the implementation process. We need high-performance hardware because a forward propagation of the CNN for a 64×64×64 voxelated volume requires around 6 GB of GPU memory; to use a batch size of 4 per GPU (i.e., processing four data pairs in parallel), at least 24 GB of memory is necessary. Furthermore, using all 8 GPUs in parallel combined with the high-speed NVLink connection reduces the average training time from 10 days (on a single A100 GPU) to 24 h for each network tested—the cascade and DRUNet usually require longer training times compared with those of DnCNN and UNet.

The networks were all trained using the Adam with weight decay regularization optimizer,^51^ with a weight decay of 0.0001 for the parameters in all layers, except for the BN parameters and bias parameters. The learning rate was scheduled with a cosine annealing learning rate,^52^ using 1000 linear warm-up mini-batch iterations to added learning stability.^53^ A batch-size of four per GPU was selected to maximize the effective use of GPU memory resources. The base learning rate was set to 0.0001. The gradient clipping value was set to 2 for BN layers and 1 for other layers to avoid exploding gradients and faster training.^54^ The optimization, data augmentation, and configuration sections of the codebase for this work were inspired by the open-source PyTorch Connectomics package^55^ for easier prototyping of the trained models.

## 3 Results

### 3.1 Denoising Performance

**Fig. 4:**
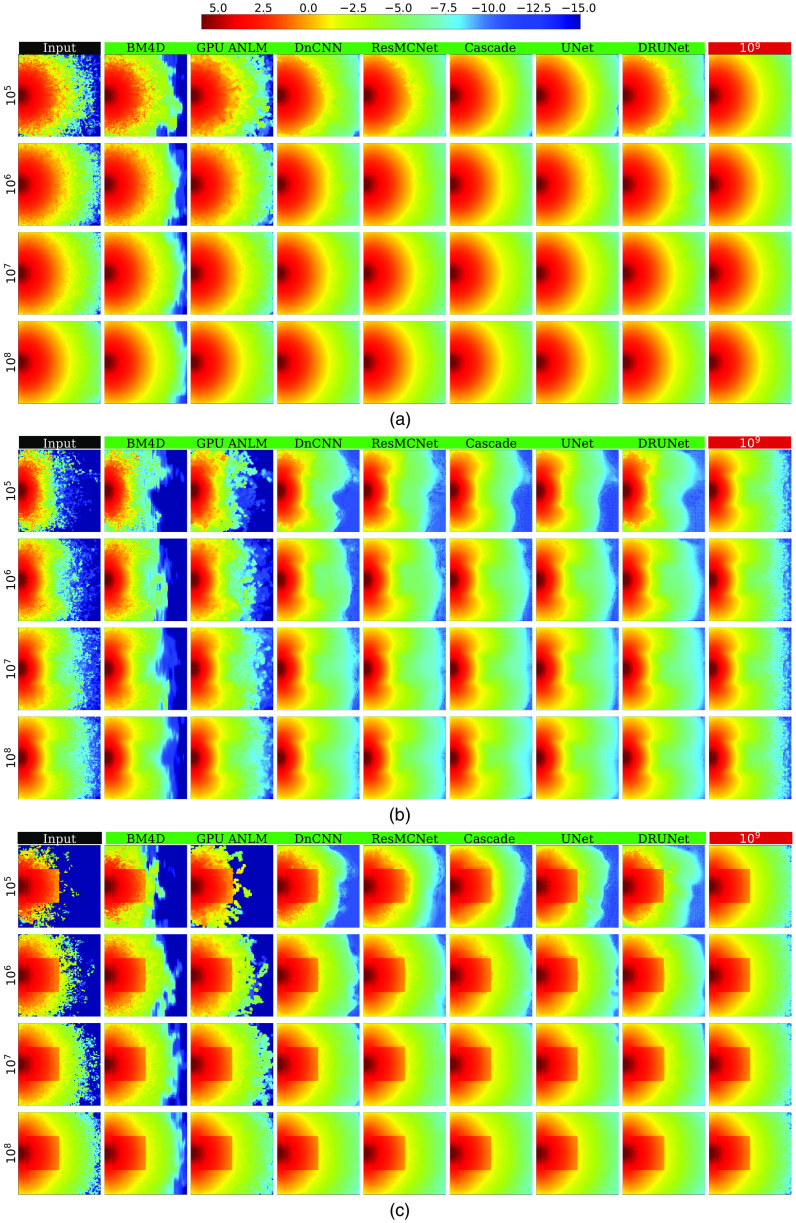
Comparisons between various denoisers in three benchmarks: (a) a homogeneous cube and the same cube containing inclusions with (b) absorption and (c) refractive-index contrasts.

**Fig. 5:**
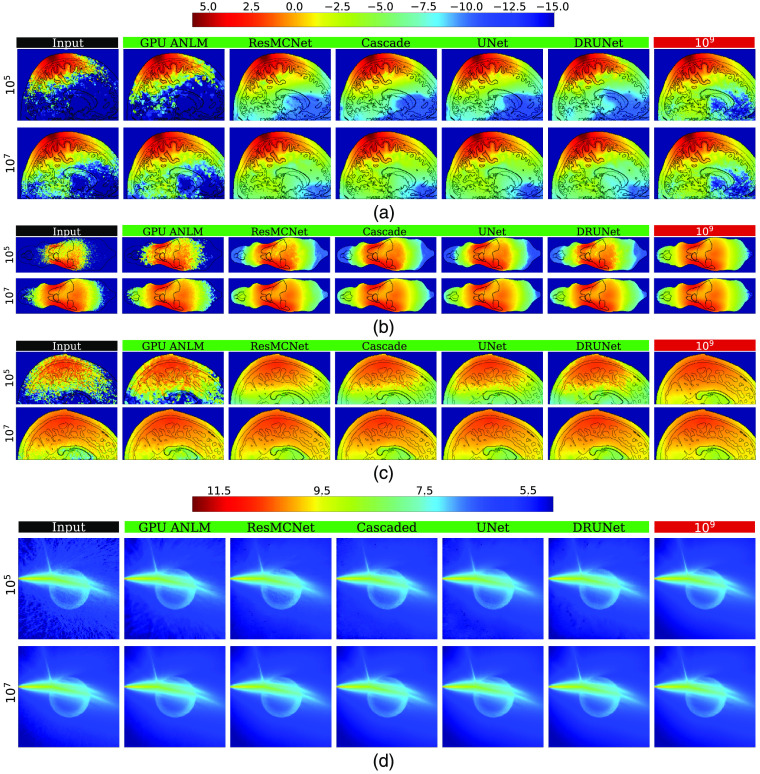
Comparisons between various denoisers in four complex benchmarks: (a) Colin27, (b) Digimouse, (c) USC-195 atlases, and (d) ball-lens benchmark.

In Fig. 4, we visually compare the fluence maps before and after denoising for each tested denoiser and photon number ($10^{5}$ to $10^{8}$) for three standard benchmarks^16^ (B1, B2, and B3). Table 1 summarizes the global metrics derived from the outputs of each denoiser; computed local metrics including mean ΔSNR and $M_{F}$ are given in Table 3. Each entry in both tables is averaged from 100 independently seeded repeated simulations. In both tables, the best-performing metrics are highlighted in bold. Similarly, a visual comparison between those from more complex domains, including Colin27, Digimouse, USC-19.5 atlases, and the ball-lens benchmark, are shown in Fig. 5. The corresponding global metrics are summarized in Table 2. Due to limited space, in Fig. 5, we only show representative images with $10^{5}$ and $10^{7}$ photons, and we removed DnCNN and BM4D due to their relatively poor performance. For the same reason, BM4D global metric results were removed from Table 2.

**Table 1:** Average global metrics derived from three basic benchmarks: (B1) a homogeneous cube, the same cube with (B2) as absorption, and (B3) refractive index inclusion; each data point was averaged over 100 repetitions. The best performing models are highlighted in bold.

		Cascade			UNet			ResMCNet			DnCNN		
Metric		B1	B2	B3	B1	B2	B3	B1	B2	B3	B1	B2	B3
MSE	$10^{5}$	**0.0031**	**0.0071**	**0.0192**	0.0050	0.0087	0.0210	0.0066	0.0114	0.0224	0.0121	0.0245	0.0507
	$10^{6}$	0.0005	**0.0009**	0.0036	0.0012	0.0017	0.0047	**0.0004**	0.0010	**0.0028**	0.0008	0.0017	0.0044
	$10^{7}$	0.0001	0.0002	**0.0007**	0.0001	0.0003	0.0012	0.0001	0.0002	0.0009	0.0002	0.0003	0.0008
SSIM	$10^{5}$	**0.9472**	**0.9063**	**0.8663**	0.9194	0.8776	0.8343	0.8741	0.8731	0.8345	0.8154	0.8131	0.7679
	$10^{6}$	**0.9690**	**0.9769**	**0.9670**	0.9117	0.9520	0.9541	0.9680	0.9600	0.9472	0.9610	0.9392	0.9174
	$10^{7}$	0.9891	0.9839	0.9817	0.9757	0.9580	0.9594	0.9930	**0.9893**	**0.9851**	0.9924	0.9876	0.9812
PSNR	$10^{5}$	**57.1445**	**53.5681**	**49.2250**	55.0538	52.7036	48.8291	53.8926	51.5285	48.5567	51.2112	48.1769	44.9994
	$10^{6}$	65.4709	**62.2454**	56.5081	61.3193	59.6665	55.2916	**65.6337**	61.9344	**57.5427**	62.9706	59.7418	55.6253
	$10^{7}$	70.9347	68.5446	**63.4404**	70.5365	67.4766	61.1240	71.3430	69.4779	62.3238	69.5030	67.9231	62.9773
		DRUNet			GPU-ANLM			BM4D					
		B1	B2	B3	B1	B2	B3	B1	B2	B3			
MSE	$10^{5}$	0.0105	0.0244	0.0400	0.0830	0.0752	0.1009	0.2223	0.1826	0.4005			
	$10^{6}$	0.0005	0.0013	0.0037	0.0130	0.0132	0.0163	0.0395	0.0383	0.3475			
	$10^{7}$	**0.0001**	**0.0002**	0.0011	0.0017	0.0018	0.0022	0.0051	0.0056	0.3366			
SSIM	$10^{5}$	0.8223	0.8115	0.7728	0.6745	0.7579	0.7092	0.5676	0.7102	0.5802			
	$10^{6}$	0.9578	0.9355	0.9227	0.8463	0.8555	0.8304	0.7174	0.7790	0.6130			
	$10^{7}$	**0.9933**	0.9877	0.9829	0.9627	0.9521	0.9459	0.8945	0.8862	0.6605			
PSNR	$10^{5}$	51.8499	48.1884	46.0353	42.8553	43.2828	42.0060	38.5718	39.4263	36.0164			
	$10^{6}$	65.1219	61.0106	56.3168	50.8978	50.8340	49.9111	46.0782	46.2030	36.6318			
	$10^{7}$	**71.6660**	**69.6645**	61.6879	59.8368	59.4459	58.6816	54.9554	54.5706	36.7706			

**Table 2:** Average global metrics derived from four complex benchmarks: (B4) Colin27, (B5) Digimouse, (B6) USC-195 atlases, and (B7) ball-lens; each data point was averaged over 100 repetitions. The best performing models are highlighted in bold.

		Cascade				UNet				ResMCNet			
Metric		B4	B5	B6	B7	B4	B5	B6	B7	B4	B5	B6	B7
MSE	$10^{5}$	**0.0121**	**0.0701**	0.0192	0.1948	0.0121	0.0741	**0.0179**	0.1451	0.0134	0.0878	0.0197	**0.0218**
	$10^{6}$	**0.0019**	**0.0072**	**0.0025**	**0.00087**	0.0020	0.0073	0.0027	0.00109	0.0022	0.0097	0.0028	0.00147
	$10^{7}$	**0.0005**	**0.0011**	**0.0007**	**0.00019**	0.0006	0.0013	0.0007	0.00020	0.0009	0.0027	0.0011	0.00038
SSIM	$10^{5}$	0.9325	**0.8893**	0.9193	0.6936	0.9311	0.8884	0.9187	0.6604	**0.9333**	0.8795	**0.9213**	**0.7824**
	$10^{6}$	**0.9699**	**0.9548**	**0.9588**	0.9864	0.9668	0.9464	0.9574	0.9861	0.9670	0.9373	0.9580	0.9616
	$10^{7}$	**0.9875**	**0.9800**	0.9825	0.9943	0.9833	0.9702	0.9807	0.9943	0.9870	0.9765	**0.9825**	0.9895
PSNR	$10^{5}$	**51.2382**	**43.5914**	49.2199	39.1466	51.2260	43.3460	**49.5335**	40.4247	50.7901	42.6124	49.1071	**48.6613**
	$10^{6}$	**59.3005**	**53.4987**	**58.0114**	**62.6631**	58.9310	53.4007	57.7991	61.6850	58.5947	52.1780	57.6404	60.3685
	$10^{7}$	**64.8770**	**61.6786**	**63.8532**	**69.2028**	64.4113	60.8132	63.6941	68.9366	62.6247	57.7291	61.8291	66.2240
		DnCNN				DRUNet				GPU-ANLM			
		B4	B5	B6	B7	B4	B5	B6	B7				
MSE	$10^{5}$	0.0162	0.1314	0.0243	0.1058	0.0159	0.1246	0.0233	0.0368	0.0667	0.3393	0.0724	0.0474
	$10^{6}$	0.0022	0.0115	0.0028	0.00139	0.0023	0.0119	0.0028	0.00094	0.0407	0.1860	0.0383	0.00435
	$10^{7}$	0.0006	0.0014	0.0007	0.00022	0.0007	0.0020	0.0007	0.00030	0.0325	0.1244	0.0283	0.00066
SSIM	$10^{5}$	0.9221	0.8676	0.9111	0.5448	0.9213	0.8747	0.9118	0.7395	0.8995	0.8379	0.8941	0.5903
	$10^{6}$	0.9608	0.9291	0.9508	0.9584	0.9583	0.9307	0.9492	**0.9892**	0.9290	0.8756	0.9210	0.8612
	$10^{7}$	0.9846	0.9737	0.9791	0.9920	0.9843	0.9734	0.9792	**0.9952**	0.9615	0.9214	0.9542	0.9696
PSNR	$10^{5}$	49.9522	40.8572	48.1905	41.7980	50.0399	41.0909	48.3797	46.3841	43.8036	36.7362	43.4453	45.2894
	$10^{6}$	58.5953	51.4205	57.5059	60.6077	58.5107	51.2805	57.5798	62.3347	45.9432	39.3454	46.2047	55.6566
	$10^{7}$	64.2162	60.6277	63.5614	68.4772	63.9421	59.1151	63.7079	67.2629	46.9164	41.0927	47.5314	63.8789

**Table 3:** Overall average SNR improvements ($\Delta {\mathrm{SNR}}_{\mathrm{all}}$ in dB) and those ($\Delta {\mathrm{SNR}}_{\mathrm{eff}}$) in the effective region (where ΔSNR>0.5  dB) as well as the photon number multipliers ($M_{F}$) in the three basic benchmarks (B1–B3).

		Cascade			UNet			ResMCNet			DnCNN		
Metric		B1	B2	B3	B1	B2	B3	B1	B2	B3	B1	B2	B3
${\Delta \mathrm{SNR}}_{\text{all}}$	$10^{5}$	**18.9565**	**14.4447**	**14.0396**	17.9565	13.8928	12.4257	16.5492	14.0211	13.9645	14.0433	10.1034	10.8670
	$10^{6}$	16.1272	**15.6349**	**14.5478**	15.3836	15.1638	14.3808	14.8762	14.5573	14.0718	12.6780	11.5296	11.6779
	$10^{7}$	14.2469	**14.5437**	11.9179	13.8348	14.5078	**12.2426**	12.2592	13.1425	11.2820	11.6208	12.4844	9.8576
${\Delta \mathrm{SNR}}_{\mathrm{eff}}$	$10^{5}$	**19.9450**	**15.8887**	**15.0143**	18.1420	14.4981	13.3353	16.9265	14.6245	14.4381	15.2666	12.2980	12.1903
	$10^{6}$	17.5282	**16.9855**	15.9910	17.6826	16.8447	**16.5500**	15.6969	15.3844	14.8730	14.8391	12.8447	13.0727
	$10^{7}$	16.1786	16.3347	14.1783	**16.3152**	**16.7415**	**14.7926**	13.3403	14.3993	12.5291	13.0195	14.1350	11.2339
$M_{F}$	$10^{5}$	**78.6412**	**27.8272**	**25.3490**	62.4669	24.5064	17.4812	45.1773	25.2412	24.9144	25.3706	10.2409	12.2096
	$10^{6}$	40.9940	**36.6008**	**28.4957**	34.5430	32.8382	27.4208	30.7341	28.5581	25.5376	18.5268	14.2220	14.7160
	$10^{7}$	26.5883	**28.4689**	15.5521	24.1813	28.2345	**16.7595**	16.8236	20.6182	13.4338	14.5238	17.7190	9.6774
		DRUNet			GPU-ANLM			BM4D					
		B1	B2	B3	B1	B2	B3	B1	B2	B3			
${\Delta \mathrm{SNR}}_{\mathrm{all}}$	$10^{5}$	16.7708	11.3500	10.8442	3.4626	1.8360	3.4951	0.3025	0.0764	1.6269			
	$10^{6}$	**17.1190**	12.3108	13.3963	3.2328	2.1149	3.3851	−0.1642	−0.7534	1.5810			
	$10^{7}$	**15.4513**	14.4728	11.9020	3.7428	2.4283	2.8556	0.5299	−0.3598	2.9538			
${\Delta \mathrm{SNR}}_{\mathrm{eff}}$	$10^{5}$	17.3218	11.7335	11.2324	4.4198	4.0586	4.5701	3.5290	2.1401	5.4370			
	$10^{6}$	**17.6998**	12.7275	13.8388	4.5288	3.4911	4.0976	2.7033	3.2510	6.0742			
	$10^{7}$	16.2252	15.0653	12.4164	4.6025	3.7708	4.1295	4.9978	3.2997	8.5494			
$M_{F}$	$10^{5}$	47.5423	13.6458	12.1456	2.2195	1.5262	2.2362	1.0721	1.0177	1.4544			
	$10^{6}$	**51.5110**	17.0247	21.8590	2.1051	1.6274	2.1803	0.9629	0.8407	1.4391			
	$10^{7}$	**35.0857**	28.0079	15.4953	2.3674	1.7492	1.9300	1.1298	0.9205	1.9741			

From the denoised images shown in Fig. 4, we can first confirm that all CNN-based denoisers show noise-adaptive capability similar to ANLM and BM4D—they apply a higher level of smoothing in noisy areas within low-photon regions and apply little smoothing in areas with sufficient photon fluence. From Fig. 4(c), we can also observe that all CNN denoisers show edge-preservation capability, again similar to ANLM and BM4D. Both noise-adaptiveness and edge-preservation are considered desirable for an MC denoiser.^16^ Because all CNN networks were trained on images of 64×64×64  voxels whereas all three benchmarks shown in Fig. 4 are 100×100×100  voxel domains, these results clearly suggest that our trained networks can be directly applied to image domain sizes that are different from the training domain size.

By visually inspecting and comparing the denoised images in Figs. 4 and 5, we observed that all CNN-based methods appear to achieve significantly better results compared with model-based denoising methods (BM4D and GPU ANLM); such difference is even more pronounced in low-photon simulations ($10^{5}$ and $10^{6}$ photons). Although the CNN denoisers were trained on shapes with less complexity, the images in Fig. 5 indicate that they are clearly capable of denoising novel structures that are significantly complex, yielding results that are close to the respective ground truth images. However, we also observe that the denoiser’s ability to recover fluence maps varies depending on the photon level in the input data—in areas where photons are sparse, the denoisers understandably create distortions that deviate from the ground truth. This can be seen clearly in the results of the complex benchmarks B4 to B7 at $10^{5}$. Nevertheless, these distorted recovered areas are still significantly better estimates than the input in the same area without denoising.

**Fig. 6:**
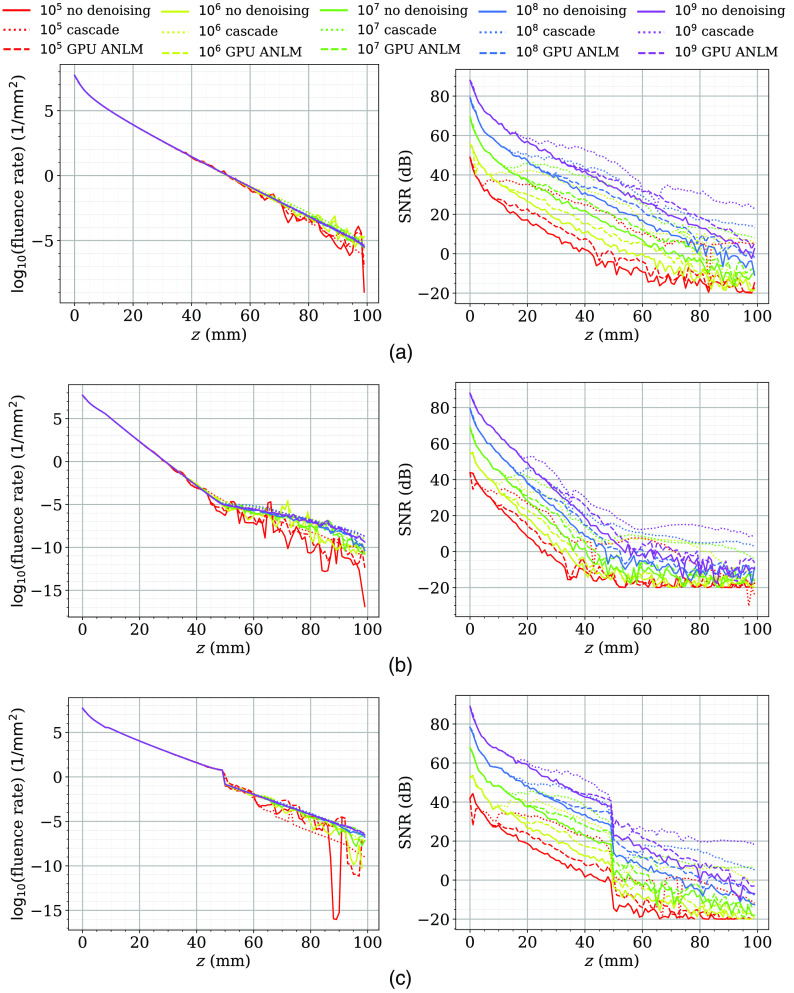
Plots of the means (left) and SNRs (right) before (solid) and after denoising using cascade network (dotted) and GPU-ANLM (dashed) in 3 benchmarks (a) B1, (b) B2, and (c) B3 along a cross section.

To confirm that CNN denoisers can produce unbiased images, the means and SNRs from benchmarks B1, B2, and B3 along the line x=50 and y=50 were calculated and plotted in Fig. 6. For brevity, we only report the results from the cascade network as representative of all CNN methods in this plot. These plots confirm that the cascade method does not alter the mean fluence of the simulations over the plotted cross section, while providing a consistent SNR improvement across a wide range of photon numbers. It also demonstrates the adaptiveness of CNN denoisers in that SNR improvement starts to decline in areas with high fluence value (thus lower noise due to shot-noise). The ∼12-dB SNR improvement shown by denoising simulations with $10^{9}$ photons (purple dotted lines over purple solid lines in the SNR plots) indicate that the cascade denoiser is capable of further enhancing image quality even it was not trained using simulations with more than $10^{9}$ photons. Such an SNR improvement is not as high as that reported from low-photon simulations, yet it is still significantly higher than the best SNR improvement produced using the GPU ANLM denoiser (dashed lines) of all tested photon numbers.

Our earlier observation that most CNN-based denoisers outperform model-based denoisers (GPU ANLM and BM4D) is also strongly evident by both the global metrics reported in Table 1 and the local metrics reported in Table 3. Among all tested CNN filters, the cascade network offers the highest performance in all tests with $10^{5}$ photons and comes close to the best performer, ResMCNet, among the $10^{6}$ test sets. Among the $10^{7}$ photon levels, DRUNet is a strong performer, with ResMCNet and cascade coming close to or surpassing it in some cases. Among the real-world complex domain benchmarks shown in Table 2, cascade reports the best performance in almost all cases, with UNet performing slightly better on USC-195 with $10^{5}$ photons and ResMCNet giving better SSIM results.

From Table 3, we can observe that all CNN-based denoisers appear to offer a five- to eightfold improvement in SNR enhancement compared with our previously reported model-based GPU ANLM filter^16^; our cascade network reports an overall SNR improvement between 14 to 19 dB across different benchmarks and photon numbers. This is equivalent to running 25× to 35× more photons in heterogeneous domains, and nearly 80× more photons for the homogeneous benchmark (B1). In other words, applying our cascade network for an MC solution with $10^{5}$ photons can obtain a result that is equivalent to running $∼2.5\times 10^{6}$ photons. In fact, except for DnCNN, the majority of our tested CNN-based denoisers can achieve a similar level of performance.

### 3.2 Assessing Equivalent Speedup Enabled by Image Denoising

In Table 4, we report the average runtimes (in seconds) of MC simulation and denoising (i.e., inference for CNN denoisers). Each test case runs on a single NVIDIA A100 with 40 GBs of memory with over 100 trials, and the time needed to transfer data between the host and the GPU is included. As we mentioned in Sec. 2.2, to obtain every denoised image, we apply CNN inference twice to handle the high dynamic range in the input data.

**Table 4:** Average runtimes (in seconds) for MC forward simulations ($T_{MC}$) and denoising ($T_{f}$) across all benchmarks, measured on an NVIDIA A100 GPU. The runtimes include memory transfer operations.

Benchmark		B1	B2	B3	B7	Colin27 (B4)	Digimouse (B5)	USC 195(B6)
Domain size		100×100×100				181×217×181	190×496×104	166×209×223
MC ($T_{\mathrm{MC}}$ [s])	$10^{5}$	0.34	0.34	0.31	0.25	0.57	0.54	0.57
	$10^{6}$	0.66	0.67	0.44	0.27	1.16	0.72	1.07
	$10^{7}$	1.93	1.91	1.14	0.40	2.76	1.49	2.56
	$10^{8}$	10.70	10.72	6.59	1.69	13.66	7.26	12.00
	$10^{9}$	87.01	87.58	55.65	14.61	105.60	58.87	90.36
Denoising ($T_{f}$ [s])	Cascade	0.27				2.93	12.08	3.06
	UNet	0.11				2.91	12.08	3.07
	ResMCNet	0.37				2.77	15.55	3.01
	DnCNN	0.19				1.41	8.76	1.50
	DRUNet	0.34				2.23	10.27	2.39
	GPU-ANLM	0.22				0.55	0.69	0.60

Table 3 suggests that, on average, about a 20 to 30 photon multiplier ($M_{F}$) is to be expected for most CNN denoisers, meaning the denoised simulations will have 20 to 30 times more photons than its input. Therefore, our goal is to identify cases in which the sum of the runtime of the baseline MC simulation running on N photons, $T_{\mathrm{MC}}(N)$, and that of the denoiser ($T_{f}$) is shorter than an MC simulation running $M_{F}\times N$ photons, i.e., $T_{\mathrm{MC}}(N)+T_{f}<T_{\mathrm{MC}}(M_{F}\times N)$. Due to space limitations, we are unable to list all combinations of simulations that satisfy the above condition. However, our general observations include the following: (1) the CNN inference runtime is independent of the number of simulated photons; (2) DnCNN is typically faster than other CNN denoisers, but it also has the poorest performance among them from Table 3; (3) the larger the domain size is, the longer it takes for CNN denoisers to run; and (4) generally speaking, applying CNN denoisers to simulations with $10^{7}$ photons or above can result in a significant reduction of total runtime.

In our previous work,^16^ we had also concluded that $10^{7}$ photon is a general threshold for GPU-ANLM to be effective; however, from the runtime data reported here using NVIDIA A100 GPUs, GPU-ANLM appears to also benefit simulations with $10^{6}$ photons, likely due to the high computing speed of the GPU. Nonetheless, comparing to most tested CNN denoisers, the GPU-ANLM denoiser offers dramatically less equivalent acceleration despite its fast speed.

## 4 Conclusion

In summary, we have developed a framework for applying state-of-the-art DL methods for denoising 3D images of MC photon simulations in turbid media. A list of supervised CNN denoisers, including DnCNN, UNet, ResMCNet, and DRUNet, were implemented, extended for processing 3D data, and tested for denoising MC outputs. In addition, we have developed a customized cascaded DnCNN/UNet denoiser combining the global-noise removal capability of DnCNN and local-noise removal capability of UNet. All developed MC denoising networks were trained using GPU accelerated MCX simulations of random domains to learn the underlying noise from MC outputs at a range of photon numbers. A simple yet effective synthetic training data generation approach was developed to produce complex simulation domains with random inclusions made of 3D polyhedral and ASCII characters with random optical properties and simulation parameters. In addition to following current best practices of contemporary CNN and DL development, we have also specifically fine-tuned and customized our MC denoisers to better handle the unique challenges arising in denoising 3D MC data. For example, to handle the high dynamic range in MC fluene maps using CNNs, a reversible log-mapping scheme was applied to each volume before being fed to the models. In addition, we have also applied inference twice and combined the results to further enhance the dynamic range of the input data. All reported CNN MC denoisers have been implemented in the Python programming language using the PyTorch framework, with both source codes and training data freely available to the community as open-source software.

To evaluate the efficacy of these proposed CNN denoisers, we have constructed seven standard benchmarks—three simple domains and four complex ones—from which we have derived and reported both global performance metrics (such as SSIM and PSNR) and local performance metrics (such as ΔSNR and $M_{F}$). From our results, all tested CNN-based denoisers offered significantly improved image quality compared with model-based image denoisers such as GPU-ANLM and BM4D in this particular application. Overall, most CNN denoisers provide a 10- to 20-dB SNR improvement on average, equivalent to running 10- to 100-fold more photons. Among these CNN denoisers, our proposed cascade network outperformed most of the state-of-the-art spatial domain denoising architectures and yielded the best image quality for low-photon simulations with $10^{5}$ and $10^{6}$ photons. Its performance is on-par with or only slightly inferior to DRUNet in high-photon simulations ($10^{7}$ photon) in simple domain tests. For all benchmarks involving real-world complex domains, the cascade network yielded the highest global metrics in nearly all tests. In comparison, some of the most effective model-based image denoisers such as the GPU-ANLM filter that we proposed previously^16^ only yielded 3 to 4 dB improvement, despite being relatively fast to compute. It is worth noting that the cascade network yielded an impressive 80-fold equivalent speedup when processing low-image-feature simulations such as a homogeneous domain.

From our tests, CNN denoisers demonstrate superior scalability to input data sizes and input image qualities. Although our training data were produced on a 64×64×64 voxelated space with relatively simple shapes, all tested CNN denoisers show no difficulty in handling images of larger sizes or significantly more complex inclusions. Our cascade network also reported a 12-dB average SNR improvement when being applied to denoise baseline simulations with $10^{9}$ photons—the level of photon number that was used as the ground truth for training. This suggest that these CNN denoising architectures may not be strictly limited by the quality of the data that they are trained on.

From our results on runtimes, most CNN denoiser inference (including two passes) time ranges between less than a second to a dozen seconds, regardless of the input data quality. We concluded that, to yield an overall shorter total runtime, applying CNN denoisers to processing MC images generated from $10^{7}$ photons or more can generally lead to significantly improved computational efficiency.

One of the limitations of the current work is the relatively long training time. To train each denoising network using our synthetic dataset of 1500 random domains (each with five photon number levels with multiple rotated views) requires on average a full day (24 h) if running on a high-end 8-GPU server with large-memory NVIDIA A100 GPUs (40 GB memory allows for using a batch-size of 4 for acceleration). If running on a single GPU node, we anticipate that the required training time is around 10 to 12 days on a single A100 GPU and even longer for low-memory GPUs. Experimenting with the number of layers in each model to reduce the number of intermediate tensors while retaining the performance benefits reported in this work, as well as the development of new and significantly more compact DL-based denoisers, will be the focus of our future work. Moreover, some of the training parameters were determined empirically and deserve further optimization. For example, we trained the networks over 64×64×64 domains. It will be significantly faster if we can reduce the training data size while still retaining the scalability to arbitrarily sized domains. Additionally, the landscapes of CNN architecture and denoising networks are constantly being updated and improved over the past few years. We cannot exhaust all emerging CNN denoisers and would be happy to extend this work with newer and more effective CNN denoising architectures in the future.

To conclude, we strongly believe that investigating high-performance image denoising techniques offers a new direction for researchers seeking for the next major breakthrough in speed acceleration for MC simulations. DL- and CNN-based image denoising techniques have demonstrated impressive capabilities compared with the more traditional model-based denoising methods and have yielded notable image quality enhancement that is equivalent to running 10 to 50 times more photons, which can be directly translated to a 10- to 50- fold speedup, in most of our tested benchmarks. Our cascade denoising network even reported a nearly 80-fold equivalent speedup when denoising homogeneous domain results—a level of acceleration that we were only able to witness when migrating MC from single-threaded computing to massively parallel hardware over a decade ago.^11^^–^^13^ With the combination of advanced image processing methods and new simulation techniques, we anticipate that MC will play an increasingly important role in today’s biomedical optics data analysis and instrument development.

## Code, Data, and Materials Availability

All software and data generated from this work, including our Python implementations of various CNN denoisers and scripts to recreate the training/testing datasets, are freely available to the research community as open-source software and can be downloaded at http://mcx.space.
